# Usability and ease of use of long-term remote monitoring of physical activity for individuals with acquired brain injury in community: a qualitative analysis

**DOI:** 10.3389/fnins.2023.1220581

**Published:** 2023-09-13

**Authors:** Marie Mazzeo, Gabriel Hernan, Akhila Veerubhotla

**Affiliations:** Department of Rehabilitation Medicine, New York University - Grossman School of Medicine, New York, NY, United States

**Keywords:** stroke, traumatic brain injury, fitness trackers, community integration, usability, ease-of-use, remote patient monitoring

## Abstract

**Introduction:**

Objective and continuous monitoring of physical activity over the long-term in the community is perhaps the most important step in the paradigm shift toward evidence-based practice and personalized therapy for successful community integration. With the advancement in technology, physical activity monitors have become the go-to tools for objective and continuous monitoring of everyday physical activity in the community. While these devices are widely used in many patient populations, their use in individuals with acquired brain injury is slowly gaining traction. The first step before using activity monitors in this population is to understand the patient perspective on usability and ease of use of physical activity monitors at different wear locations. However, there are no studies that have looked at the feasibility and patient perspectives on long-term utilization of activity monitors in individuals with acquired brain injury.

**Methods:**

This pilot study aims to fill this gap and understand patient-reported aspects of the feasibility of using physical activity monitors for long-term use in community-dwelling individuals with acquired brain injury.

**Results:**

This pilot study found that patients with acquired brain injury faced challenges specific to their functional limitations and that the activity monitors worn on the waist or wrist may be better suited in this population.

**Discussion:**

The unique wear location-specific challenges faced by individuals with ABI need to be taken into account when selecting wearable activity monitors for long term use in this population.

## 1. Introduction

Acquired brain injury (ABI) is an umbrella term that describes damage to the brain that occurs after birth; mechanisms of injury may be traumatic [e.g., traumatic brain injury (TBI)] or non-traumatic (e.g., stroke). ABI is a significant cause of morbidity and mortality worldwide, the recovery course is extremely varied, and residual disability is highly prevalent (Törnbom et al., 2017; Grabljevec et al., 2018). Potential sequelae include impaired functioning in physical, cognitive, neurological, behavioral, and lifestyle domains and most of these limitations persist even into the chronic phase (>6 months) post-ABI. Compared to individuals with similar durations of hospitalization for different etiologies, individuals who have experienced a head injury have an increased risk of death for at least 13 years after hospital admission (Hillman et al., 2008).

Rehabilitation is an important part of the recovery process post-ABI and typically continues during the acute phase and the chronic phase. An essential aspect of rehabilitation during the chronic phase is physical activity (PA). Rehabilitation specialists are recommended to prescribe PA programs, especially in the chronic phase after ABI (Grabljevec et al., 2018). Increased physical activity has been associated with distinct anatomical and physiological changes and may improve physical and mental health; aerobic activity has demonstrable benefits on overall brain health (Hillman et al., 2008; Crosson et al., 2017; O'Carroll et al., 2020; Mercier et al., 2021; Sheng et al., 2021). PA is believed to facilitate neuronal plasticity and affect the brain’s recovery following ABI, and engagement in PA has been shown to impact an individual’s health-related quality of life (Hillman et al., 2008; Crosson et al., 2017; O'Carroll et al., 2020; Mercier et al., 2021; Sheng et al., 2021). Additionally, engagement in physical activity may improve sleep quality; sleep disturbances are highly prevalent in individuals with ABI and have a well-established impact on the efficacy of rehabilitation efforts (Bruijel et al., 2021; Dey et al., 2021). The significant role of PA in recovery is especially significant when considering that recent studies have demonstrated that individuals who have experienced ABI participate in less PA compared to healthy individuals, and are more sedentary than their age-matched peers (Törnbom et al., 2017).

Traditionally, questionnaires and surveys are used to measure PA; however, wearable physical activity monitors (PAMs) have emerged as an alternative objective method to measure PA (Brickwood et al., 2019; Cho et al., 2021; Veerubhotla et al., 2021). PAMs are designed to be small, lightweight, and low-cost devices (Brickwood et al., 2019; Veerubhotla et al., 2021). Wearable devices are beneficial because they can monitor PA over days to months in free-living conditions with minimal interference to the user’s everyday life (Brickwood et al., 2019; Veerubhotla et al., 2021). The data derived from PAMs can be described as person-generated health data (PGHD)—a potentially valuable resource for researchers and care providers alike (Cho et al., 2021; Veerubhotla et al., 2021). For example, accelerometer counts have been used to measure walking intensity in individuals who have experienced a minor stroke and are significantly associated with physical capacity, a measure of functional status related to overall health and well-being (Braakhuis et al., 2022).

Usability and wearability are important considerations for the use of PAMs. As described by Eng et al., usability refers to the ease of use, which encompasses user interface, set-up, and errors (Louie et al., 2020). Wearability refers to donning, doffing, aesthetics, and the comfort of a device (Louie et al., 2020). A recent systematic review identified user-related factors (e.g., device non-wear) and device/technical-related factors (e.g., issues with hardware, software, etc.) as major categories that impact the quality of PGHD (Cho et al., 2021). It is essential that PAMs are specifically studied in individuals who have experienced ABI, because the unique sequelae which impact the usability and accuracy of these devices in this patient population may not be represented in studies of the general population (Campos et al., 2018; Veerubhotla et al., 2021). Additionally, to the best of our knowledge, the transition from laboratory to community-based studies of PAMs has not yet occurred for individuals who have experienced TBI (Veerubhotla et al., 2021). Further, there is a need for usability studies and community-based research in both individuals with TBI and stroke that have a duration of greater than 1 week (Hardy et al., 2018; Veerubhotla et al., 2021). There is a lack of information regarding the long-term utilization of PAMs, nor is there a consensus on the best wear location for PAMs, as studies have used different wear locations (e.g., wrist, waist, ankle) (Giggins et al., 2017).

To effectively undertake community-based research in individuals with ABI using wearable devices, it is important to first understand their usability and ease of use from the patient/user perspective. The goal of this pilot investigation was to determine the usability and feasibility of wearable PAM in individuals with ABI, specifically, in individuals who have experienced a stroke or TBI. This study assessed patient-reported challenges with PAMs at three popular wear locations (wrist, waist and ankle), patient reported wear location preferences, and the use of a remote data transfer hub for remote monitoring, for a duration of 4 weeks in community dwelling individuals with ABI. By doing so, this study seeks to provide a framework for important considerations for the use of PAMs in community-dwelling individuals who have experienced ABI.

## 2. Materials and methods

### 2.1. Participants

To be included in this study, participants had to (1) be between the ages of 45 and 75; (2) have been diagnosed with a stroke or a non-penetrating TBI by a physician and be at least 6 months post-injury; (3) have been medically stable for 3 months at the time of study participation; (4) no plans to make any drastic changes to medications for at least 4 weeks; (5) have sufficient endurance and motor ability to ambulate 10 m continuously with minimal assistance; (6) willing and able to give informed consent, and (7) be able and willing to comply with study procedures and verbal instructions.

Individuals were excluded from participation in this study if they had (1) existing severe cardiac conditions such as myocardial infarction or congestive heart failure; (2) fluctuating blood pressure; (3) a history of uncontrolled seizure disorder; (4) additional orthopedic, neuromuscular, or neurological conditions that would interfere with the ability to perform the assessments; (5) difficulty following or responding to commands that would limit the study participation, and (6) enrollment in another research study or therapy at the time of starting this study.

Individuals were recruited via telephone using a convenience sample from Kessler Foundation and the Kessler Institute for Rehabilitation (KIR) System. Of the 12 participants who attended the initial visit, two participants declined to participate, citing the 4-week time commitment.

All participants were paid $25 for the initial visit to Kessler Foundation and $50 for each week they completed study procedures in the community.

### 2.2. Design and procedure

The ActiGraph GT9X Link (ActiGraph LLC, FL, United States) was the physical activity monitor chosen for this study. The ActiGraph GT9X Link is an FDA-approved class II medical device that weighs 14 grams, has dimensions of 3.5 × 3.5 × 1 cm, and saves movement data without any identifiable information related to participants (User Guide ActiGraph GT9X Link + ActiLife, 2020). The ActiGraph PAM is widely used in physical activity research across patient populations and is considered a gold standard for physical activity outcomes.

**Figure 1:**
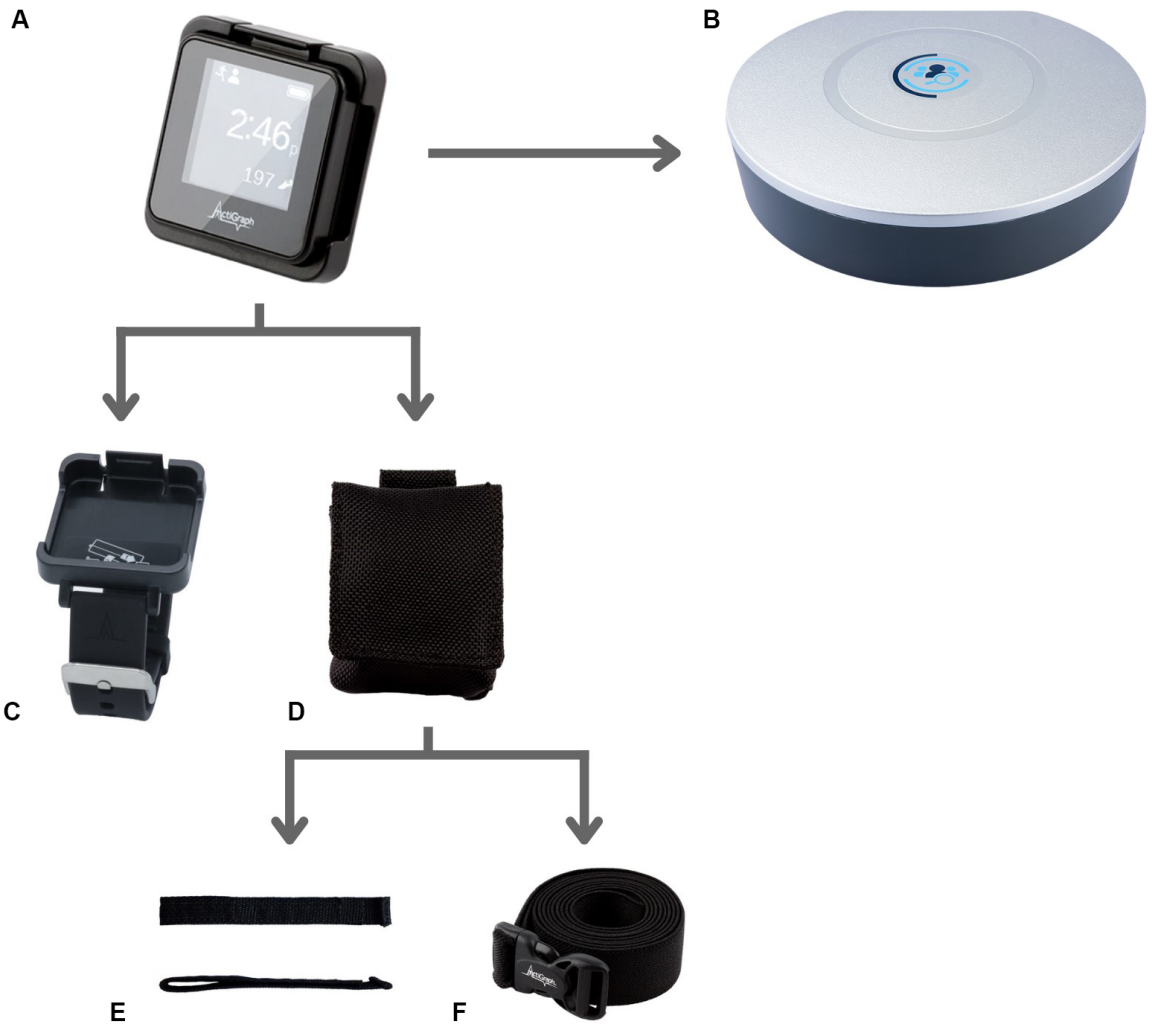
PAM and related study accessories. **(A)** ActiGraph GT9X Link PAM worn simultaneously by all study participants on the wrist, waist and ankle. **(B)** CentrePoint Data Hub used by all study participants for remotely transferring the daily PA data to the research server. **(C)** Wrist watch accessory for ActiGraph GT9X Link PAM used to wear the PAM on the wrist. **(D–F)** Pouch accessory, **(D)** used along with the ankle Velcro strap, **(E)** accessory and waist belt, **(F)** accessory to wear the PAM on the ankle and waist, respectively.

Following informed consent, participants were provided with three ActiGraph Link physical activity monitors and associated accessories, which included the dock and charger, CentrePoint Data Hub, sensor pouch, wrist band, ankle strap, and waist belt (Figure 1). All participants were trained to wear, charge, and dock the physical activity monitors for automatic remote data transfer to the research ActiGraph server using the dock and Data Hub. Participants were sent home with a detailed instruction sheet with instructions and graphical representation of instructions covered during the training. Participants were asked to go about their everyday routine in the community as usual and were not asked to engage in any physical activity specifically for the study in the community. Participants were instructed to wear all three ActiGraph activity monitors (one each on their non-affected or dominant wrist, ankle and waist) simultaneously for 4-weeks in the community and try to wear the activity monitors simultaneously for at least 10 hours during their wake time each day. Participants were instructed to charge each activity monitor once each week for a minimum of 2 hours.

This study follows the consolidated criteria for reporting qualitative research (COREQ) checklist. The questionnaires and semi-structured interviews, organized by wear location, were administered by AV (Ph.D., post-doctoral fellow, female) and OI (BS, research assistant, male). Neither AV nor OI had any relationship with the participants before the study commencement. During the initial visit, participants were introduced to the interviewers and their credentials. Study procedures, aims, and goals were discussed during informed consent. At the end of each week, AV or OI contacted the participant via telephone. Individuals were not specifically asked about the presence of non-participants (e.g., family members at home) during these phone calls.

Each week, participants were asked to complete a System Usability Scale (SUS) and an After-Scenario Questionnaire (ASQ). The SUS was used to collect feedback regarding wear locations; it is a 10-item questionnaire with a five-response option Likert scale; it is well-validated and commonly utilized in usability research (Bangor et al., 2009; Klug, 2017). The ASQ was used to quantify the perceived usability of remote data transfer; it is a 3-item questionnaire with a seven-response option Likert scale, it is popular in usability studies due to its simplicity (Lewis, 1991).

Semi-structured interview questions were not provided to participants in advance; however, repeat interviews with the same questions were carried out weekly for 4 weeks. The interview duration was approximately 5–10 min. Field notes were made during the interview, and transcripts were not returned to participants for comment or correction. Data saturation was not discussed with participants.

At the end of the 4-week study period, participants were asked to rank preferred wear locations and return the study equipment. Participants were not asked to provide feedback on the findings.

### 2.3. Research ethics

Written informed consent was obtained from all participants before enrollment. All study procedures involving human subjects were approved by the Institutional Review Board at Kessler Foundation, Protocol Number: R-1141-21.

## 3. Analysis

### 3.1. System usability scale

The SUS is composed of 10 alternating positive and negative statements (Bangor et al., 2009; Klug, 2017). Odd-numbered questions are scored 0–4, and even-numbered questions are scored 4–0 (Bangor et al., 2009; Klug, 2017). The sum of the scores yields a value between 0 and 40, this is subsequently multiplied by 2.5 to generate a SUS score out of 100. The numerical value can then be converted into a letter grade (Bangor et al., 2009; Klug, 2017). The 4 weeks of SUS scores for each participant were averaged for incorporation into the final analysis. Calculations were performed using Microsoft Excel.

### 3.2. After scenario questionnaire

The ASQ is calculated by using the average of the response to the three questions; if missing values were present, they were discarded. Higher scores reflect better usability, lower scores represent that the participants felt unsatisfied with either the ease of completing the tasks, the amount of time it took, and the support information, or a combination of these three (Lewis, 1991). The 4 weeks of ASQ scores for each participant were averaged for incorporation into the final analysis. Calculations were performed using Microsoft Excel.

### 3.3. Semi-structured interviews

Semi-structured interviews were analyzed using the Framework Method of Content Analysis, which consists of well-defined steps; this makes it a popular method in qualitative analysis, especially in health research (Goldsmith, 2021). There are five key steps: (1) data familiarization, (2) identifying a thematic framework; (3) indexing all study data against the framework; (4) charting to summarize the indexed data; (5) mapping and interpretation of patterns found within the charts (Bryman and Burgess, 1994; Goldsmith, 2021).

Deidentified transcript data were compiled and organized by a Participant ID. Researchers MM (4th-year medical student, research assistant, female) and GH (undergraduate, research assistant, male) familiarized themselves with the documents by reading the full transcriptions. Initial themes were derived directly from the semi-structured interview questions. Additional information provided which did not fit into the initial categories was added to a “Miscellaneous/Other Group.” After initial coding, themes were refined by MM and GH, and agreed upon by AV; data categorization was discussed to ensure each final code represented participant responses. This process was done by hand.

Additionally, MM and GH assessed each transcript to determine whether the participants’ opinions leaned positive, negative, or neutral/ambivalent. MM and GH discussed and agreed upon each participant’s emotional valence (Table 1).

**Table 1:** Emotional valence and PAM ranking.

	Wrist	Ankle	Waist
**S1**	Positive**(1)**	Ambivalent**(3)**	Positive**(2)**
**S2**	Positive**(1)**	Negative**(2)**	Negative**(3)**
**S3**	Negative**(3)**	Negative**(2)**	Positive**(1)**
**S4**	Ambivalent**(1)**	Ambivalent**(2)**	Negative**(3)**
**S5**	Negative**(3)**	Ambivalent**(2)**	Positive**(1)**
**TBI 1**	Ambivalent**(1)**	Negative**(3)**	Ambivalent**(2)**
**TBI 2**	Ambivalent**(1)**	Ambivalent**(2)**	Ambivalent**(3)**
**TBI 3**	Ambivalent**(1)**	Ambivalent**(2)**	Ambivalent**(3)**
**TBI 4**	Ambivalent**(1)**	Ambivalent**(3)**	Ambivalent**(2)**
**TBI 5**	Ambivalent**(3)**	Ambivalent**(1)**	Ambivalent**(2)**

## 4. Results

The final pilot included 10 participants: 5 who had experienced a stroke, and 5 who had experienced a TBI (Table 2). The average age of the participants was about 60 years. None of the study participants used a wearable “activity monitor” or “fitness tracker” or “smart watch” before participating in this study. All of the participants who attended and completed the initial visit and started wearing PAM in the community completed the full 4-week duration of the study. Of note, two individuals in the TBI group lost a sensor (one wrist and one ankle). The study team replaced lost sensors by new sensors and the participants were asked to continue the study. Three of the five individuals with stroke wore the activity monitor on their right wrist and ankle (non-affected side or dominant side) while two of the five individuals with TBI wore the activity monitor on their right wrist and ankle (non-affected or dominant side). All other participants wore the activity monitor on their left wrist and ankle.

**Table 2:** Study participant demographics.

	Stroke	TBI
Participants	*n* = 5	*n* = 5
Female	*n* = 1	*n* = 2
Male	*n* = 4	*n* = 3
Mean age	64.2	56.4
Age range	62–65	49–64

### 4.1. Usability questionnaire

**Figure 2:**
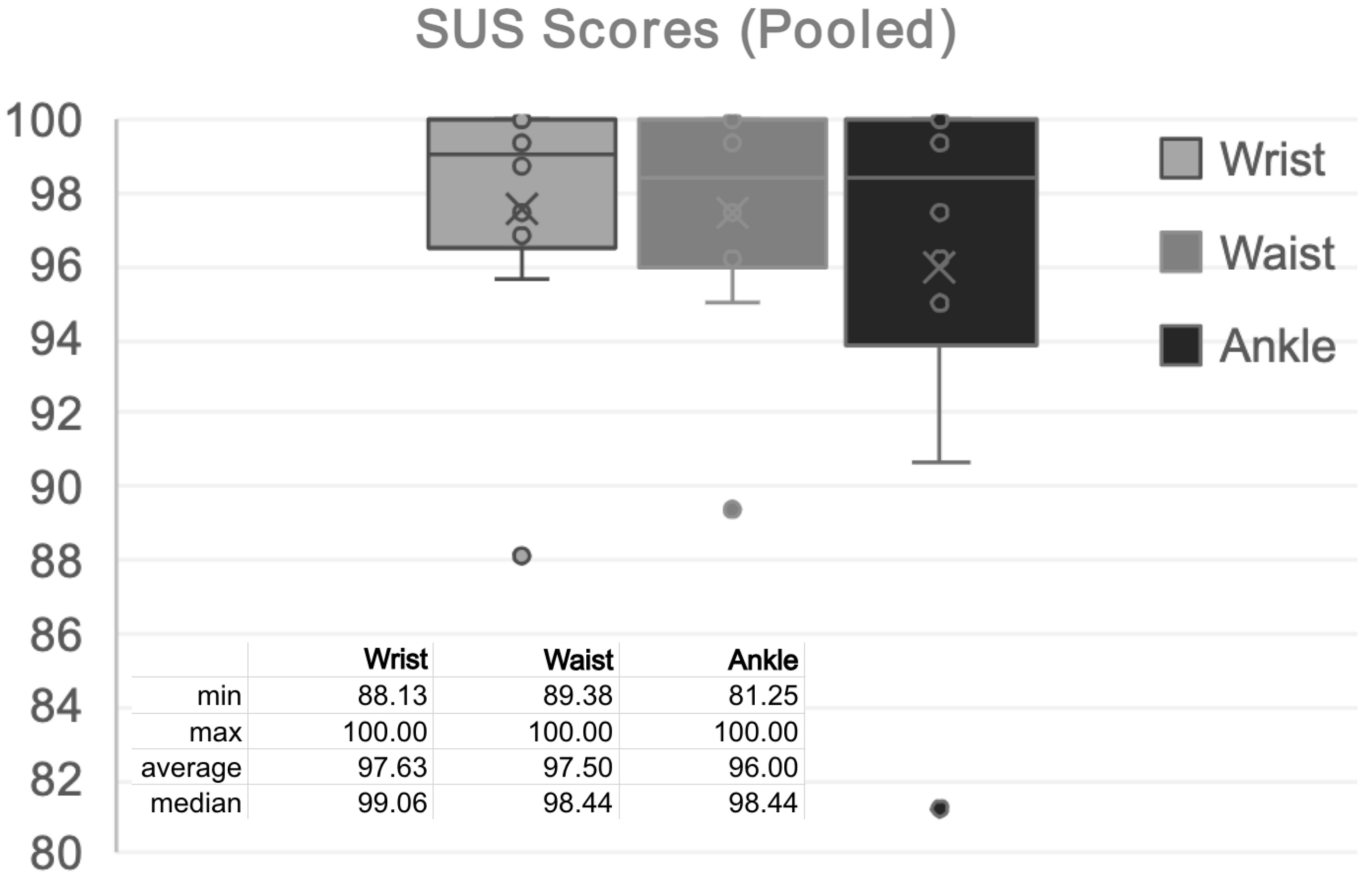
Pooled SUS scores.

Average pooled SUS Score for PAM placement was 97.63 for the wrist (Standard Deviation, SD: 3.68), 97.50 for the waist (SD: 3.37), and 96 for the ankle (SD: 6.01) (Figure 2). The average pooled ASQ Score for remote data transfer was 1.033 (SD 0.11).

### 4.2. Qualitative analysis

The major themes were “no challenges,” location-specific usability and wearability, and location-independent general impressions are shown in Table 3.

**Table 3:** Qualitative analysis: major and minor themes.

	Major themes	Minor themes
Wrist	“No challenges”	*Donning/Doffing*
		*Daily routine*
	Usability	*Functional limitations impact use*
		*Intuitive*
		*Task-specific challenges*
	Wearability	*Comfort*
Ankle	“No challenges”	*Donning/Doffing*
		*Daily routine*
	Usability	*Functional limitations impact use*
		*Requires effort/Assistance*
Wearability	*Discomfort*
		*Public perception*
Waist	“No challenges”	*Donning/Doffing*
		*Daily routine*
	Usability	*Functional limitations impact use*
		*Easy*
	Wearability	*Adjust for comfort*
General impressions	Accessory impressions/Suggestions	*Strap material*
		*Sensor/Pouch preferences*
	User interface	*Question utility*
		*Technical challenges*

#### 4.2.1. Wrist usability and wearability

##### 4.2.1.1. “No challenges”

Three participants in the stroke group reported “no challenges” with donning and doffing the wrist monitor. One participant reported difficulty with both actions; the other participant reported difficulty donning the device but could doff without assistance. Both of the individuals reported receiving help from family members to address these challenges. All participants in the TBI group reported “no challenges” in donning or doffing the device; however, one participant added that it was easier to “put on” than “take off” the PAM. All participants reported “no challenges” when asked about difficulty going about their daily routine.

##### 4.2.1.2. Functional limitations impact use

Two participants in the stroke group reported functional limitations in one of their hands that impacted the use of the wrist monitor. One of these individuals reported difficulty with the wrist monitor all 4-weeks, stating, “It’s slightly challenging to take it off given the functionality in my hands… I need two functional hands to take off the sensor from the wrist strap.” On week two, the other participant reported occasional difficulty donning the wrist monitor, and stated that they “sometimes have limited function in one hand.” This participant was able to doff the monitor without assistance.

##### 4.2.1.3. Intuitive use

Of the participants who made additional comments regarding the wrist monitor, “intuitive use” emerged as a minor usability theme. Participants often compared these devices to wearing a watch. As one participant in the stroke group stated, “[The wrist monitor] is easy to wear, like a wristwatch, I wear one every day, I cannot function without a watch; it’s the most memorable routine.”

##### 4.2.1.4. Task-specific challenges

One participant in the stroke group and one participant in the TBI group described task-specific challenges. The participant in the stroke group stated, “Yes, [there is some difficulty] while doing some mechanical work, if I need to get my hand somewhere, it pops off and gets in the way. I’m trying to be more careful.” The participant in the TBI group described, “[There is some difficulty] when at work, I’m handling babies. The wrist sensor is bulky, and I’m afraid it might get in my way of work. I’m used to wearing my watch on my left wrist and not my right. I’m being extra cautious at work and tucking the sensor under my sleeve.”

##### 4.2.1.5. Comfort

Two participants in the TBI group made additional comments regarding the comfort of this device; one stated, “It’s snug, I really do not feel it,” and the other described it as “comfortable.”

#### 4.2.2. Ankle usability and wearability

##### 4.2.2.1. “No challenges”

Three participants in the stroke group reported “no challenges” with donning and doffing the ankle monitor. These challenges were addressed by utilizing the assistance of a family member. All of the participants in the TBI group reported “no challenges” with donning and doffing the ankle monitor. Three participants in the stroke group, and all of the participants in the TBI group, reported “no challenges” going about their daily routine while wearing the ankle monitor.

##### 4.2.2.2. Functional limitations impact use

Of the two participants in the stroke group that reported a functional limitation in their hands, both reported difficulty with the ankle monitor. One participant utilized the assistance of a family member, and the other replaced the velcro strap with an elastic pull-on band. One participant in the stroke group stated, “Yes, I had a lot of difficulties [with the ankle monitor]. It is almost impossible to put on with one hand.”

##### 4.2.2.3. Requires effort/Assistance

Of the participants who made additional comments regarding the ankle monitor, “Requires Effort/Assistance” emerged as a minor usability theme, this included requiring assistance with donning/doffing the device, finding the device when it fell out of the pouch, and keeping conscious tabs on device. One participant in the stroke group stated, “The Velcro does not hold for too long. I had to take it off at the golf course; I did not realize I had lost the activity monitor until another group of people playing behind us came and asked if it belonged to any one of us.” A similar sentiment was described by a participant in the TBI group, “…it did fall off when I was at work… I looked around and asked people, and someone found the sensor for me. Now I keep a conscious tab on the sensor.”

##### 4.2.2.4. Discomfort

Of the participants who made additional comments regarding the ankle monitor, “Discomfort” emerged as a minor wearability theme. For example, one participant in the stroke group noted, “Sometimes it gets in the way when crossing my legs, but it’s alright.” Two participants in the TBI group described it was necessary to wear socks with this device to improve comfort.

##### 4.2.2.5. Public perception

One participant in the stroke group made a comment regarding public perception of the device, stating, “I just need to hide it, people ask if I’m not allowed to be outside the home because it looks like a tracker, and people ask me if I’m on house arrest. I wear long pants to hide it. When people cannot see, it’s okay, does not bother me.”

#### 4.2.3. Waist usability and wearability

##### 4.2.3.1. “No challenges”

Four participants in the stroke group reported “no challenges” with donning and doffing the waist monitor. One participant reported difficulty and addressed this challenge through the assistance of a family member. All of the participants in the TBI group reported “no challenges” with donning and doffing the waist monitor. Four participants in the stroke group, and all participants in the TBI group, reported “no challenges” going about their daily routine while wearing the waist monitor.

##### 4.2.3.2. Functional limitations impact use

Of the two participants in the stroke group that reported a functional limitation in their hands, only one reported difficulty with the waist monitor, difficulty was reported on all 4 weeks. This participant addressed this challenge through assistance from a family member.

##### 4.2.3.3. Easy

Of the participants who made additional comments regarding the waist monitor, “easy” emerged as a minor wearability theme. For example, one participant in the stroke group noted, “This is the easiest and most convenient to use.” Another participant said it was “just like wearing clothes,” and another stated, “all sensors should be like this.”

##### 4.2.3.4. Adjust for comfort

Of the participants who made additional comments regarding the waist monitor, “Adjust for Comfort” emerged as another minor wearability theme. One participant in the stroke group found that the waist monitor was “quite stable, just like wearing a waist belt,” but added that they “just need to pull it up or down” to make it comfortable. Another participant in the stroke group reported, “if [the waist monitor is] on a shirt, it feels great, but if it touches the skin, it itches slightly.” Additionally, a participant in the TBI group said that “every now and then, it has to be re-adjusted to make sure it’s hanging at the side.”

#### 4.2.4. General impressions: accessory impressions/suggestions

##### 4.2.4.1. Strap material preference

Although many of the comments regarding “Strap Material Preference” emerged in the context of specific wear locations, this was categorized as a separate theme because PAM accessories vary widely depending on the manufacturer. Comments regarding the wrist monitor are not included in this section because this accessory had a different mechanism to secure the monitor (Figure 1). One participant in the stroke group mentioned that they replaced the Velcro strap with an elastic band to “slip it on with one hand.” Two participants in the TBI group described that it would be helpful if the pouch were securely attached to the strap; as one of these participants explained, “The strap stuck and was clinging to my sock but the sensor fell off, so having a pouch such that the sensor does not fall off unless the strap also falls off from the ankle would be helpful.”

##### 4.2.4.2. Sensor/Pouch preference

Of the participants that made impressions and suggestions regarding the accessories, “sensor/pouch preference” emerged as a minor theme. One participant in the TBI group mentioned that “[the sensor] is a little bulky, but it’s ok.” Two other participants in the TBI groups spoke about the requirement for water resistance. One of these participants explained that “it would be nice if the pouch were water resistant because when it rains, and [they] step into a puddle, the pouch gets wet.” The other participant in the TBI group spoke about needing “to remember to take it off during showering and put it back on.”

#### 4.2.5. General impressions: user interface

Some participants made general impressions that were not location specific; one major theme that emerged was the user interface.

##### 4.2.5.1. Question utility

Of the participants that made impressions and suggestions regarding the user interface, “question utility” emerged as a minor theme. One participant in the stroke group explained that they “use activity monitor phone-based apps” and were not sure why they “need the activity monitors in general.”

##### 4.2.5.2. Technical challenges

Of the participants that made impressions and suggestions regarding the user interface, “technical challenges” emerged as another minor theme. One participant in the TBI group remarked that “the last 2 days it did not show the circle for sending data.” Another stated that “the sensor could not transfer data remotely” but added that once this sensor was replaced with a new sensor, it “work[ed] well.”

### 4.3. Preferred wear location

The final ranking for each of the participants demonstrates that a majority of participants (7/10) preferred the wrist sensor. Two individuals ranked the waist sensor in first place, and one individual ranked the ankle sensor in first place. Utilizing ranked-choice voting may help visualize the overall ratings. A ranking of first place received one point, second place received two points, and third place received three points. Rankings for each location were calculated: wrist rank = 16, ankle rank = 22, and waist rank = 22. Based on these results, the wrist location was the preferred location, and the waist and ankle were tied.

## 5. Discussion

This study explored the use of PAMs in community-dwelling adults who had experienced an acquired brain injury, for 4 weeks, at three wear locations: the wrist, ankle, and waist. To the best of our knowledge, this is the first study to assess the perception of PAMs for a duration greater than 1 week in two key subsets of individuals with ABI, participants who had experienced TBI (*n* = 5) and stroke (*n* = 5).

The quantitative analysis portion of this study utilized two questionnaires which are popularly used in usability research, the SUS to assess each wear location and the ASQ to evaluate the data hub for remote data transfer. Average pooled SUS Scores for each wear location received a score of an A/A+, well within the acceptability range. The average pooled ASQ was 1.033, representing that participants “strongly agree” that the data hub was easy to use. Of note, 9/10 participants provided all three ASQ categories with a score of 1, or “strongly agree,” for all 4 weeks. The remaining participant, a member of the TBI group, provided a score of 3 for Question 2: Overall, I am satisfied with the amount of time it took to complete this task for weeks one and two; however, they provided a score of 1 for the remaining 2 weeks. This improvement in rating likely represents an increase in satisfaction with the duration of time required to remotely transfer data or a learning curve on how to use the remote data hub to transfer the data. Taken together, these data support the assertion that the use of PAMs at each wear location, and remote data transfer, were user-friendly. This is a notable finding as “poor usability experience” has been identified as a factor contributing to user non-wear (Cho et al., 2021).

The major and minor themes revealed by qualitative analysis provide a deeper picture of the participants’ perspectives on these devices. Based on our investigation, the participants interviewed described the wrist sensor as one that was easy and intuitive to use and was considered comfortable; however, it interfered with specific tasks due to its bulk, such as working with machinery or holding babies. “User’s lifestyle or not wearing for certain activities” is a factor that impacts the quality of PGHD, and having a small, lightweight, and inconspicuous device has been identified as an important wearability factor (Louie et al., 2020; Cho et al., 2021). Some participants found that the ankle monitor required effort/assistance; this sensor was often dropped, and some participants checked on it throughout the day. Effort expenditure was reflected in the wearability of the ankle monitor; some participants found this device uncomfortable, either physically or psychologically (due to concerns regarding public perception). Being “unsatisfied with the appearance of the device” and “discomfort” are both factors that may contribute to user non-wear; having a “cosmetically pleasing” device has been identified as an important wearability factor (Louie et al., 2020; Cho et al., 2021). The participants’ notable comments regarding the waist monitor demonstrate that some individuals favored this sensor, and found it the most comfortable, though it did require adjustment for some individuals.

Two participants described functional limitations in their hands and had difficulty donning and doffing the PAMs. Although these comments emerged regarding all three placement locations, it is notable many of these comments were regarding the wrist sensor. As demonstrated in Figure 1, the wrist sensor has a watch-buckle design. Fewer comments were made regarding the waist and ankle sensors, which do not have this buckle design. The requirement for accessible design features, especially in this patient population, is supported by the literature. “User’s health condition prevents device use” has been identified as a factor that affects the quality of PGHD (Cho et al., 2021). Additionally, a focus group comprised of individuals with stroke and physical therapists identified the ability to don and doff a PAM with one hand as an important feature of wearable technology (Louie et al., 2020).

Many of the participants had negative perceptions of the ActiGraph GT9X Link strap accessories. Some participants believed that having a Velcro scrap would improve usability and wearability for the wrist worn PAM. A commonly cited difficulty was that the sensor pouch was not securely attached to the ankle strap (Figure 1); of note, this was not a problem for the wrist sensor, though this device had distinct difficulties. Notable comments for improvement regarding the sensor itself included addressing its bulk and the desire for this device to be waterproof. A waterproof sensor would allow individuals to participate in daily activities without having to consider removing the device and having to remember it afterward. “Forget to wear” has been identified as a component of user non-wear (Cho et al., 2021). Of note, the ActiGraph GT9X Link is water-resistant (IP27) for up to 1 meter and up to 30 min (User Guide ActiGraph GT9X Link + ActiLife, 2020). However, as raised by one of the participants, the pouch provided with the ankle monitor was not water resistant.

Device and technical-related factors have also been identified as factors that affect PGHD (Cho et al., 2021). A few participants noted some technical difficulty with the device, though these issues were infrequent and did not affect the perceived usability based on the results of the SUS and ASQ questionnaires. Another consideration raised by a participant is the utility of these devices compared to smart phone-based apps. The requirement for a wearable PAM vs. a smartphone app has been raised in the literature; a study of 21 chronically ill people found that some participants preferred using a cell phone app over a GPS tracking watch; however, further research is required to evaluate the efficacy of phone-based devices in individuals who experienced ABI (Hardy et al., 2018). Regardless, this raises an important consideration regarding the significance of user preferences when considering the incorporation of PAMs in clinical practice.

The emotional valence determination (ambivalent, lean positive, or lean negative) derived from the transcripts by MM and GH further highlights the significance of personal preferences in PAM location selection. The first-place ranking by two individuals, positive emotional valence for two individuals, and waist usability theme of “easy” supports the assertion that some individuals may prefer a waist sensor to a wrist or ankle sensor. This is an important consideration for health care professionals considering integrating PAMs into their rehabilitation practice.

This study has some limitations. Primarily, the sample size of this pilot study was small (*n* = 10). Additional limitations include a lack of demographic data, the presence of any lasting deficits (other than those elicited during the semi-structured interview), employment information, functional assessments and information on injury sequelae. An additional limitation of this study is that the semi-structured interview was of short duration, which resulted in limited transcripts.

## 6. Conclusion

This pilot investigation contributes to the literature regarding considerations for PAM wear location, patient preferences, and challenges specific to this population. Following an ABI, individuals report difficulty participating in, and sustaining, physical activity (Törnbom et al., 2017). PAMs may be a motivating factor for engaging in physical activity (McClure, 2002; Lynch et al., 2018). Additionally, as this technology continues to improve and access to PAMs becomes easier and affordable, these devices have a variety of benefits that may be helpful in this patient population. For example, in addition to the objective data collection on gait and fitness, the Apple Watch may detect falls and alert emergency contacts, and service providers, that help is required; this research is currently ongoing (Strauss et al., 2021). The incorporation of wearable PAM in the chronic phases of rehabilitation following ABI has the potential to provide valuable benefits for patients, caretakers, researchers, and rehabilitation professionals. However, to improve the usability and increasing the incorporation of PAM in longitudinal studies in individuals with ABI the challenges specific to this population need to be taken into account when choosing PAM and wear location.

## Data availability statement

The de-identified data supporting the conclusions of this article will be made available by the authors, upon reasonable request.

## Ethics statement

The studies involving humans were approved by the Institutional Review Board at Kessler Foundation. The studies were conducted in accordance with the local legislation and institutional requirements. The participants provided their written informed consent to participate in this study.

## Author contributions

MM was a medical student mentored by AV and was involved with data analysis and drafting the manuscript. GH was an undergraduate student volunteer mentored by AV and was involved in data analysis and drafting the manuscript. AV was the principal investigator of this study and designed the study, collected data and was involved in data analysis and editing the manuscript. All authors contributed to the article and approved the submitted version.

## Funding

This study was funded by the New Jersey Health Foundation (Grant # PC 10–21).

## Conflict of interest

The authors declare that the research was conducted in the absence of any commercial or financial relationships that could be construed as a potential conflict of interest.

## Publisher’s note

All claims expressed in this article are solely those of the authors and do not necessarily represent those of their affiliated organizations, or those of the publisher, the editors and the reviewers. Any product that may be evaluated in this article, or claim that may be made by its manufacturer, is not guaranteed or endorsed by the publisher.
